# Evaluating physicochemical, nutritional and sensory properties of plant-based surimi: A focus on protein source

**DOI:** 10.1016/j.fochx.2025.103257

**Published:** 2025-11-04

**Authors:** Shahriyar Valizadeh, Armin Mirzapour-Kouhdasht, Mahagani Lasciers, Reza Tahergorabi

**Affiliations:** a Food and Nutritional Sciences Program, North Carolina Agricultural and Technical State University, Greensboro, NC 27411, USA; b Department of Food Science, Purdue University, West Lafayette, IN 47907, USA

**Keywords:** Konjac glucomannan, Oleogel, Plant-based surimi, Protein gelation, Seafood analogs, Sustainability, Thermal properties, Texture profile

## Abstract

This study focuses on the development and characterization of plant-based surimi formulations incorporating soy, pea, and mung bean protein isolates. Seven formulations were evaluated for their physicochemical, rheological, thermal, sensory, and nutritional properties to determine their potential as alternatives to traditional fish-based surimi. Among the tested formulations, the sample prepared with pea protein isolate exhibited textural attributes most comparable to conventional surimi, which was supported by scanning electron microscopy revealing a dense and continuous protein matrix. The pea protein-based surimi also demonstrated the highest whiteness index. Sensory evaluation further indicated a preference for the pea-based formulation, particularly in terms of texture and overall acceptability. Although the protein content of the plant-based surimi was lower than that of conventional surimi, the plant-based blend outperformed in fat and ash contents. These results underscore the critical role of protein source selection in the development of nutritionally balanced and functionally viable plant-based surimi products.

## Highlights

• Plant-based surimi was made using soy, pea, and mung bean protein isolates.
• Pea protein-based surimi had greater gel strength and compact microstructure.
• Konjac glucomannan and oleogel enhanced texture, appearance, and moisture retention.

## 1 Introduction

The global food industry is undergoing a transformative shift, spurred by growing consumer awareness of health, environmental sustainability, and ethical considerations. Within this evolving landscape, surimi—a processed seafood product made from finely ground and deboned fish—has remained a staple in many cuisines for its affordability, extended shelf life, and versatile applications. Surimi's unique texture and high protein content have made it particularly appealing, with the market valued at USD 3.78 billion in 2022 and projected to grow at a compound annual growth rate (CAGR) of 6.1% through 2030 (Nugroho, Zulbainarni, Asikin, Budijanto, & Marimin, 2024). However, the traditional production of surimi is fraught with challenges, including overfishing, habitat destruction, high water consumption and low yield, which collectively threaten marine ecosystems and raise sustainability concerns (Food and Agriculture Organization (FAO), 2023). These issues have led to an urgent need for alternative, more sustainable solutions within the seafood industry.

Simultaneously, a paradigm shift in consumer behavior has driven the rapid expansion of the plant-based seafood market, valued at USD 81.4 million in 2023 and projected to grow to USD 853.7 million by 2032 with an extraordinary CAGR of 29.5%. This growth is fueled by a convergence of factors, including rising health consciousness, concerns about contaminants such as mercury and microplastics, and increasing advocacy for animal welfare (Mahmud, Islam, & Tahergorabi, 2024). The market's growth is further supported by consumer interest in plant-based proteins, with 15% of American households purchasing plant-based seafood in retail in 2023, and 62% of those households making repeat purchases. This indicates a solid consumer base and potential for future growth as product offerings expand and consumer awareness increases. Moreover, plant-based seafood offers substantial sustainability benefits: it reduces reliance on wild-caught fisheries, decreases greenhouse gas emissions, and lowers water and energy consumption compared to conventional seafood production. These environmental advantages, coupled with growing consumer demand for ethical and eco-friendly food options, underscore the urgent need to develop innovative plant-based seafood products that are nutritious, appealing, and scalable. Expanding the plant-based seafood market also presents economic opportunities, supporting new food technology ventures and contributing to a resilient and sustainable food system.

Plant-based seafood products remain scarce in the market, and limited research has been conducted on the development of plant-based surimi-style seafood. Seafood proteins are known for their high biological value, offering essential amino acids (EAAs) in a nutritionally optimal balance. In comparison, plant proteins are typically considered to have lower nutritional quality. However, combining proteins from different legume sources can result in a more complete and balanced EAA profile. Soy protein isolate (SPI) is the most refined form of soybean protein, containing over 90% protein. It is considered a high-quality plant-based protein and is among the few capable of effectively replacing animal proteins. SPI plays a key role in providing the viscoelastic textural properties essential in processed vegetarian products—such as plant-based meat alternatives. Pea protein products are reported to offer similar and complementary functional properties to their soybean counterparts; however, their heat-induced gels tend to be weaker than those formed by soy proteins. Mung beans (*Vigna radiata* L.) are a significant legume crop widely cultivated across Southeast Asia, South America, Australia, and Africa. They are nutritionally rich, containing EAA, minerals, and vitamins B and C. Mung bean protein isolate (MBPI) is not only more easily digestible than other legume proteins but also relatively cost-effective. Due to these advantages, MBPI is gaining interest as a promising ingredient for plant-based meat products (Liu et al., 2021). However, its use in food applications remains limited because of inferior functional properties specifically, the gelation ability compared to SPI (Ratnawati, Desnilasari, Surahman, & Kumalasari, 2019).

Traditional surimi products are made using heat-induced gelation, which enables the formation of various gel-based textures. Protein gelation involves a three-dimensional structural transformation—often linked to changes in surface hydrophobicity—along with modifications in electrostatic interactions, the formation of cross-links, or a combination of these factors (Oyom, Awuku, Faraji, Bi, & Tahergorabi, 2025). Various techniques have been employed to enhance protein gelation, including different cooking methods, the use of transglutaminase to form covalent cross-links between or within proteins, heat treatment to promote disulfide bond formation, and the incorporation of plant proteins with polysaccharides like alginates. Ran, Lou, Zheng, Gu, and Yang (2022) successfully developed plant-based fish ball analogues using SPI, effectively replicating the texture of traditional fish balls by incorporating a water-soluble polysaccharide extracted from konjac (*Amorphophallus konjac*) tubers called konjac glucomannan (KGM). KGM forms thermally irreversible elastic gels under alkaline conditions. KGM enhances texture, chewiness, and water-holding capacity and has been widely used in meat and seafood analogs to improve gel strength and overall sensory acceptability (Shrestha, Van’t, Haritos, & Dhital, 2023).

To provide an alkaline environment for KGM in plant-based surimi, sodium carbonate (Na₂CO₃) is added to promote deacetylation of KGM, which strengthens intermolecular associations and forms a thermally stable elastic gel network, thereby enhancing hardness, resilience, and water-holding capacity. Liu et al. (2019) reported that Na₂CO₃ accelerates gelation in maize starch–*Mesona chinensis* polysaccharide gels by lowering the pasting or denaturation temperature, facilitating protein unfolding and cross-linking.

Sucrose also contributes to gelation and texture by stabilizing protein–polysaccharide matrices through hydrogen bonding and reduced water activity. Yang, Yang, and Yang (2018) demonstrated that sucrose enhances gelation temperature, gel strength, and network density of κ-carrageenan gels by promoting tighter molecular packing and limiting water mobility.

Unlike fish-based surimi, which naturally contains omega-3 fatty acids, plant-based surimi lacks these essential nutrients. To address this nutritional gap, various oil sources can be considered, including flaxseed oil and vegetable oils such as sunflower oil (Ran, Yang, Chen, & Yang, 2022). Sunflower oil is particularly rich in linoleic acid, comprising 48–74% of its total fatty acids, but contains negligible amounts of linolenic acid compared to seed oils like soybean and rapeseed. It also includes trace levels of very long-chain saturated fatty acids such as arachidic and behenic acids (Salas, Bootello, & Garcés, 2015). Flaxseed oil, on the other hand, is valued for its high α-linolenic acid (ALA) content, typically exceeding 45%. Yet the conversion of ALA to long-chain omega-3 fatty acids such as EPA and DHA in humans is inefficiently less than 1% for DHA—making flaxseed oil a poor substitute for the marine-derived omega-3s required to achieve comparable health benefits (Shim, Gui, Wang, & Reaney, 2015). In contrast, algae oil (*Schizochytrium* sp.) offers a sustainable, plant-based source of EPA and DHA, providing nutritional benefits similar to fish. With its neutral flavor, high smoke point, and eco-friendly production process, algae oil represents a valuable alternative for enriching plant-based surimi without compromising taste or functional properties (Nowacka et al., 2023).

Recent studies suggest that directly adding liquid oil can hinder protein cross-linking, weaken the gel properties of surimi, and increase the risk of oxidative deterioration, ultimately compromising product quality (Shen et al., 2024; Zhang et al., 2023). As a result, exploring innovative methods for incorporating liquid oil is essential to improve its stability and dispersion in surimi gels while tailoring texture to meet specific needs. Oleogel is a semi-solid system in which liquid oil is trapped within a 3D gel network formed by oleogelators, giving the oil a gel-like consistency without changing its chemical composition. Our previous studies demonstrated that incorporating oil in the form of an oleogel enhances both the oxidative stability and texture of the fish ball and chicken nuggets (Islam, Mahmud, Oyom, Aminzare, & Tahergorabi, 2024; Mahmud, Islam, & Tahergorabi, 2024; Oyom et al., 2024; Mahmud, Islam, & Tahergorabi, 2024; Adrah, Adegoke, Nowlin, & Tahergorabi, 2022; Mahmud et al., 2023).

Whiteness is a key quality attribute in surimi seafood, as it greatly influences consumer perception and acceptance. However, formulations containing soy protein often display a yellowish tint, which can detract from their visual appeal. To address this, titanium dioxide (TiO₂) was incorporated in this study to enhance and brighten the white color of the product. In addition to its whitening effect, TiO₂ also exhibits antimicrobial properties that may contribute to extended shelf life (Sha & Xiong, 2020). Although TiO₂ has been banned for food use in the European Union, it remains approved as a color additive exempt from certification under FDA regulations (21 CFR 73.575) when used within specified limits (≤1% by weight of the food) in the United States, where this study was conducted (FDA, 2024). Its effectiveness in improving the visual appearance of plant-based products was previously demonstrated by Liao, Li, and Tjong (2020) in plant-based chicken formulations. The main objective of this study was to explore the potential of plant-based proteins—specifically soy, pea, and mung bean protein isolates, as well as their strategic blends—for developing high-quality plant-based surimi seafood. By integrating KGM and oleogel incorporation to improve texture and oxidative stability, and employing multi-level analyses including rheology, differential scanning calorimetry (DSC), scanning electron microscopy (SEM), amino acid profiling, and sensory evaluation, this study offers novel mechanistic insights and practical guidance for producing high-quality, nutritionally balanced, and visually appealing plant-based seafood analogs.

## 2 Materials and methods

### 2.1 Materials

Grade A frozen Alaska pollock surimi was sourced from Trident Seafoods Corp. (Seattle, WA). The surimi was pre-treated with cryoprotectants, including 4% sorbitol, 4% sucrose, 0.15% sodium tripolyphosphate, and 0.15% tetrasodium pyrophosphate. Each frozen surimi block weighed approximately 10 kg and was shipped overnight in heavily insulated industrial-grade containers packed with ice to maintain low temperatures. Upon arrival, the blocks were portioned, vacuum-sealed, and stored at −80 °C until further use. SPI was purchased from Bulk Supplements (Henderson, NV, USA), containing 90.0% protein, 0% fat, and 1% carbohydrate. MBPI was purchased from Green Boy Protein (Los Angeles, CA, USA), containing 80.0% protein, 3% fat, and 1% carbohydrate. Pea protein isolate (PPI) was purchased from Carlyle Nutritionals LLC (Melville, NY, USA), containing 80.0% protein, 4% fat, and <1% carbohydrate. KGM powder was purchased from Bulk Supplements (Henderson, NV, USA). β-Sitosterol and γ-oryzanol were purchased from TCI America (Portland, OR, USA). Sodium carbonate (Na₂CO₃), sea salt, tapioca starch and sucrose were purchased from Verno Goods LLC (Elizabeth NJ, USA). TiO₂ was purchased Spectrum Chemical MFG Crop, (Gardena, CA, USA) and algae oil (*Schizochytrium sp*.- contains 1000 mg DHA and 10 mg EPA) was purchased from Source-Omega, LLC, (Chapel Hill, NC, USA).

### 2.2 Plant based surimi preparation

A series of preliminary experiments was conducted to identify suitable plant proteins for developing plant-based surimi seafood. The results indicated that soy, pea, and mung bean proteins were capable of producing products with appearance and texture somewhat resembling traditional fish-based surimi. Based on these findings, a more comprehensive study was designed to optimize the formulation conditions. Various protein concentrations were tested to determine whether it was possible to match the protein content of conventional surimi while also achieving similar textural properties. Plant-based surimi preparation was conducted based on the method described by Ran, Yang, et al. (2022) with slight modifications. In this study, seven plant-based surimi (PBS) formulations were developed using different combinations of SPI, PPI, and MBPI. PBS1 contained 9% SPI, PBS2 contained 9% PPI, and PBS3 included a combination of 4.5% SPI and 4.5% PPI. PBS4 was formulated with 9% MBPI, while PBS5 contained 4.5% SPI and 4.5% MBPI. PBS6 consisted of 4.5% PPI and 4.5% MBPI, and PBS7 incorporated a balanced mixture of 3% SPI, 3% PPI, and 3% MBPI. Each protein was individually hydrated with 30% distilled water for 30 minutes. After hydration, other ingredients including sea salt (1.5%, w/w), sodium carbonate (Na₂CO₃, 0.5%, w/w), titanium dioxide (TiO₂, 0.5%, w/w), oleogel (1%, (w/w), which contained β-sitosterol and γ-oryzanol as oleogelators, and algae oil), and sucrose (0.5%, w/w) were added to all formulations. Ice was incorporated to maintain consistency and prevent heat buildup during mixing. The mixture was blended for 1 minute and 30 sec using a universal food processor (Model UMC5, Stephan Machinery Corp., Columbus, OH) at low speed which was equipped with a temperature monitoring probe and a double-layer cooling jacket. Based on preliminary tests and relevant literature (Ran, Lou, et al., 2022; Zhang, Kobata, et al., 2023), the concentration of KGM was set at 6% and gradually introduced during blending. High-speed chopping under vacuum conditions (0.5 bar) was performed for 3 minutes. Protein pastes were prepared in 500 g batches. Stainless steel tubes (17.5 cm in length, 1.9 cm internal diameter) equipped with screw end caps were used to cook the plant-based surimi pastes. The sealed tubes were submerged in a water bath and heated at 90 °C for 15 minutes. After heating, they were immediately cooled in an ice slush. The plant-based surimi gels were then removed from the tubes for subsequent analysis.

### 2.3 Surimi preparation

The preparation of surimi paste followed the procedure outlined by Sharaf Eddin, Adegoke, Ibrahim, and Tahergorabi (2021). Frozen surimi was thawed at 4 °C for 24 hours prior to processing. The thawed surimi was initially chopped at low speed for 1 minute using a universal food processor (Model UMC5, Stephan Machinery Corp., Columbus, OH, USA), equipped with a temperature monitoring probe and a double-layer cooling jacket. Sea salt (2%) was then added and chopping continued at low speed for an additional 30 seconds to form the base paste. Final moisture content was adjusted to 79% by incorporating ice along with standard functional additives. One percent tapioca starch and polyphosphate at 0.24% (Kena FP-28, Innophos, Cranbury, NJ), in dry powder forms were included during chopping. These concentrations were selected based on prior studies demonstrating their effectiveness in promoting fish protein gelation and achieving texture properties similar to commercial surimi-based seafood (Williams, Mikell, Chetachukwu Adegoke, & Tahergorabi, 2020; Sharaf Eddin, Adegoke, Issa, Wilson, & Tahergorabi, 2020; Anyanwu, Alakhrash, Hosseini, Ibrahim, & Tahergorabi, 2017; Alakhrash, Anyanwu, & Tahergorabi, 2016). The mixture was then chopped at high speed under vacuum (0.5 bar) for an additional 3 minutes to improve protein extraction and homogeneity. The prepared surimi paste was packed into stainless steel tubes (17.5 cm length, 1.9 cm internal diameter) with screw end caps and cooked in a water bath at 90 °C for 15 minutes. Following thermal processing, the tubes were rapidly cooled in an ice slush, and the gels were removed for subsequent analysis. Fish-based surimi was designated as the control (C).

### 2.4 Proximate composition and pH

The AOAC (2005) standard methods were used to analyze the proximate composition of plant-based surimi and control, including moisture, protein, total fat, and ash contents. The pH meter was first calibrated with three buffers (pH 4, 7, and 10) to ensure precise measurements. Then, samples were thoroughly mixed with deionized water at a 1:10 (w/v) ratio, and their pH was measured using an Orion Star A211 pH meter (Thermo Scientific Co., USA) (Valizadeh, Naseri, Babaei, & Hosseini, 2020).

### 2.5 Texture profile analysis

The cylindrical gel samples of plant-based surimi and the control were cut to a height of 25 mm, and a diameter of 20 mm. Texture profile analysis was performed using a texture analyzer (Model TA-XT2, Texture Analyzer, Texture Technologies Corp., Scarsdale, NY, USA). The test was conducted at a speed of 1 mm/s with a compression distance of 12.5 mm (50% of the sample height). The analysis measured key textural properties, including hardness, cohesiveness, springiness, chewiness, and resilience (Islam et al., 2024).

### 2.6 Differential scanning calorimetry (DSC) analysis

The thermal characteristics of the plant-based surimi and control samples were analyzed using a thermal analyzer (TA Q100-DSC, TA Instruments, New Castle, DE, USA). Approximately 8–12 mg of each sample was precisely weighed and sealed in aluminum pans (TA Instruments), with a sealed empty pan serving as the reference. The thermal scan was conducted from −50 °C to 200 °C at a heating rate of 5 °C/min under a nitrogen atmosphere. The onset temperature (To), peak denaturation temperature (Td), and enthalpy change (ΔH) were obtained from the thermograms using Universal Analysis 2000 software (TA Instruments) (Oyom, Awuku, et al., 2025).

### 2.7 Viscoelastic properties

The viscoelastic properties of the plant-based surimi and control samples were analyzed using a magnetic bearing rheometer (TA Instruments AR-G2, New Castle, DE, USA). A concentric cylinder geometry (15.17 mm diameter - 991,036) equipped with a temperature-controlled Peltier plate was used to examine their rheological behavior. A strain sweep test was conducted at 25°C with a constant angular frequency of 1 Hz, covering a shear strain range of 0 to 300 kPa to capture both linear and non-linear viscoelastic regions. The dynamic rheological parameters measured included the storage modulus (G′) and the loss modulus (G′′) (Oyom et al., 2025).

### 2.8 Scanning electron microscopy (SEM)

The microstructure of the plant-based surimi and control samples was examined using scanning electron microscopy (SEM). Specimens were cut into dimensions of 1 × 1 × 0.25 cm, defatted using the Soxhlet extraction method, and then freeze-dried for approximately 48 hours (Labconco, Kansas City, MO, USA). The analysis focused on the crust-to-core interface. Micrographs were obtained using a Hitachi TM-1000 microscope operating at 15 kV and 100x magnification, following the procedure described by Adrah et al. (2022) with slight modifications.

### 2.9 Color measurement

The color parameters including lightness (L*) and chromaticity parameters b* (yellow– blue) and a* (red–green) were obtained according to the method described by (Valizadeh, Naseri, Babaei, Hosseini, & Imani, 2019). A Minolta colorimeter (CR-400, Konica Minolta, Osaka, Japan) was used for this analysis, with the device calibrated using a standard white tile (L = 97.43, a = 0.01, b = 1.64). Whiteness index (WI) of samples was calculated using the following formula:

(1) $$\mathrm{WI}=100-\sqrt{a{*}^{2}+b{*}^{2}+{\left(100-L*\right)}^{2}}$$

### 2.10 Amino acid profile

A total of eighteen amino acids, including aspartic acid, threonine, serine, glutamic acid, proline, glycine, alanine, valine, isoleucine, leucine, tyrosine, phenylalanine, lysine, histidine, arginine, cysteine, and methionine, were analyzed in plant-based surimi and control. The analysis was conducted using the EMSL Analytical, Inc. laboratory reference method, as outlined by Henderson and Brooks (2010).

### 2.11 Sensory analysis

Institutional Review Board (IRB) approval for the sensory evaluation was obtained from the University's IRB office under protocol number HS25-0029. A panel of ten semi-trained evaluators assessed the sensory attributes of the plant-based surimi and control, including odor, texture, and overall acceptability. The evaluation was conducted using a 9-point hedonic scale, where 9 represented the highest quality and 1 the lowest. Samples with an overall score above 7 were considered acceptable (Mahmud, Valizadeh, Oyom, & Tahergorabi, 2024).

### 2.12 Statistical analysis

All experiments were conducted in triplicate, with results presented as mean values ± standard deviation (SD) using analysis of variance (ANOVA). Differences between sample treatments were evaluated at a 5% significance level (IBM SPSS Statistics 24, USA), and Tukey's test was applied for multiple comparisons.

## 3 Results and discussions

### 3.1 Moisture content

As shown in Table 1, the highest moisture content was observed in PBS4 and PBS2 (78.00 ± 2.01% and 77.49±2.01% respectively), Notably, the control (C) exhibited a moisture content of 75.15% ± 0.99, which was significantly lower than PBS4 and PBS2 but comparable to PBS3 and PBS6 (p > 0.05), indicating that these formulations retained a similar level of hydration as traditional surimi. This agrees with Ran et al. (2022b) who reported similar moisture contents for fish-based and plant-based fish ball. Moisture content is a key factor influencing gelation, texture, and the overall processing performance of surimi-based products. However, it is not the only determinant of gel strength and textural properties. Other molecular interactions, particularly the formation of disulfide bonds, also play a critical role. According to USDA guidelines, conventional fish-based surimi typically contains moisture levels ranging from 74% to 79%, depending on processing techniques and added ingredients (Pietrowski, Tahergorabi, Matak, Tou, & Jaczynski, 2011). The moisture levels found in almost all treatments fall within this standard range, reinforcing their potential as viable surimi alternatives with comparable hydration levels to fish-based surimi.

**Table 1:** Proximate composition of surimi (control) and plant-based surimi. Data are presented as mean ± standard deviation (n=3). Different letters within the same column indicate significant differences between mean values (Tukey’s test, α = 0.05).

Samples	Moisture (g/100g)	Ash (g/100g)	Fat (g/100g)	Protein (g/100g)
**PBS1**	76.62±1.45^B^	2.52±0.10^BC^	5.71±0.44^CD^	8.33±0.35^B^
**PBS2**	77.49±1.73^AB^	1.96±0.19^BC^	6.14±0.34^BCD^	7.20±0.26^B^
**PBS3**	74.71±1.97^C^	2.23±0.28^BC^	7.23±0.70^AB^	7.53±0.60^B^
**PBS4**	78.00±2.01^A^	1.88±0.06^C^	5.05±0.18^D^	7.57±0.15^B^
**PBS5**	76.02±1.87^BC^	2.59±0.17^AB^	6.66±026^BC^	7.83±0.15^B^
**PBS6**	74.26±1.94^C^	2.11±0.35^BC^	6.22±0.31^BCD^	7.27±0.06^B^
**PBS7**	70.75±0.95^D^	3.28±0.43^A^	8.04±0.69^A^	7.60±0.10^B^
**C**	75.15±0.99^C^	1.86±0.10^C^	2.79±0.20^E^	14.67±1.53^A^

PBS1: 9% SPI; PBS2: 9% PPI; PBS3: 4.5% SPI + 4.5% PPI; PBS4: 9% MBPI; PBS5: 4.5% SPI + 4.5% MBPI; PBS6: 4.5% PPI + 4.5% MBPI; PBS7: 3% SPI + 3% PPI + 3% MBPI.

### 3.2 Ash content

There were no significant differences in ash content among the PBS samples and the control, except for PBS7, which recorded the highest ash content (3.28% ± 0.43). This increase may be attributed to the synergistic effect of combining multiple protein sources—soy, pea, and mung bean, which likely enhanced mineral retention in the final product. According to the nutrition facts provided by the manufacturer, the pea protein used contains 23% sodium, 0.5% iron, 0.6% calcium, and 2% potassium. Mung bean protein contributes 10% sodium, while soy protein provides 17% sodium, 1.5% iron, and 4% potassium. These minerals, derived from the different protein sources and additional ingredients, may have contributed to the elevated ash content in PBS7. This suggests that formulations incorporating a variety of proteins could result in higher mineral content, aligning with previous findings (Majumdar, Chowdhury, Nath, & Mondal, 2017).

### 3.3 Fat content

Alaska pollock is considered a lean fish. Rinsing is a key step in surimi production as it removes water-soluble proteins and lipids from the fish meat, resulting in surimi with very low-fat content (Sharaf Eddin et al., 2020). In the present study, the control surimi gels had a fat content of 2.79%, which aligns with our previous findings that reported 2.23% fat in Alaska pollock surimi gels without oil addition (Anyanwu et al., 2017). As expected, the fat content of the PBS samples was significantly higher than that of the control due to the inherently higher fat content of the plant-based protein sources. For example, PPI contains 4% total fat and MBPI contains 5%, according to their respective nutrition labels, while SPI contains no fat. This may explain why formulations with a higher proportion of soy, such as PBS1, had lower fat content. Additionally, to improve the fatty acid profile of the PBS formulations, 1% oleogel was added to all plant-based surimi samples, further contributing to the increased fat content. Similarly, Anyanwu et al. (2017) reported a significantly higher fat content when 0.05% flaxseed and salmon oil were added to surimi.

### 3.4 Protein content

According to the USDA, the reported crude protein content of surimi on a wet weight basis is 15.2% (Pietrowski et al., 2011). In the present study, the control sample showed a comparable crude protein content of 14.67 ± 1.53%. However, the protein content of plant-based surimi samples (PBS) was significantly lower than that of the control. Research has indicated that plant-based meat alternatives generally have lower protein content compared to animal-derived products. For example, Costa-Catala et al. (2023) demonstrated that plant-based burgers typically contain significantly lower protein levels than beef or pork products, falling below the thresholds typically associated with high-protein foods, this discrepancy arises primarily from the inherent properties of plant-based ingredients, such as legumes, grains, and vegetables, which typically have lower protein densities compared to animal-derived sources. Another factor contributing to lower protein content in plant-based meat alternatives is their complex ingredient composition, which often includes components like fiber, starches, fillers, and oils. These ingredients, intended to improve taste and texture, can further dilute the overall protein concentration (Drewnowski, Bruins, & Besselink, 2024). This lower protein content may influence product positioning, as plant-based surimi may need to be marketed differently than traditional fish-based surimi, emphasizing benefits such as sustainability, plant-based nutrition, or functional ingredients rather than high protein content.

### 3.5 pH value

**Fig 1:**
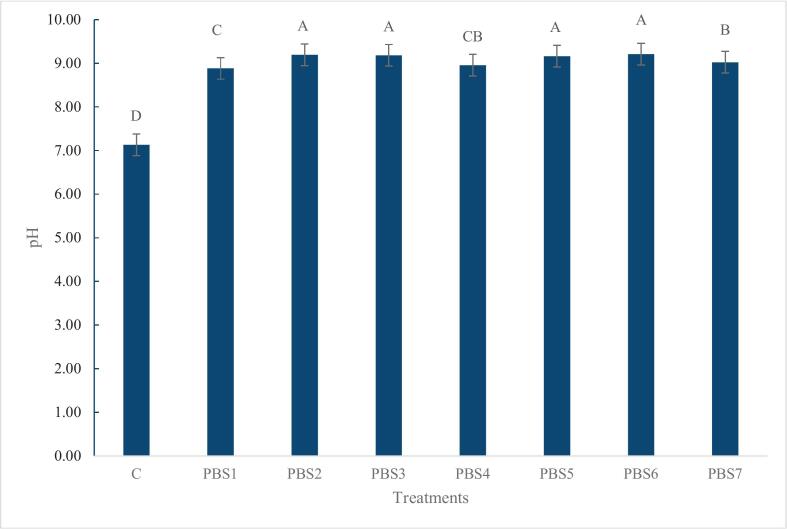
pH values of surimi (control) and plant-based surimi. Data are presented as mean ± standard deviation (n=3). Different letters above the bars indicate significant differences between mean values (Tukey’s test, α = 0.05). PBS1: 9% SPI; PBS2: 9% PPI; PBS3: 4.5% SPI + 4.5% PPI; PBS4: 9% MBPI; PBS5: 4.5% SPI + 4.5% MBPI; PBS6: 4.5% PPI + 4.5% MBPI; PBS7: 3% SPI + 3% PPI + 3% MBPI.

As shown in Fig. 1, the control sample (C) maintained a neutral pH of approximately 7.13, which is significantly lower than all the PBS samples. The plant-based surimi samples exhibited higher pH values, ranging from 8.88 to 9.21, indicating a shift towards alkaline conditions. These results are in agreement with those reported by Ran et al. (2022b).

The higher pH levels in the PBS formulations can be attributed to the addition of sodium carbonate, a known pH regulator, which diminishes steric hindrance by deacetylating KGM and promotes the formation of a robust gel network. This network is stabilized through non-covalent bonds, such as hydrogen and ionic bonds, with hydrogen bonds playing a key role. Additionally, the pH-induced anionic groups can modify water behavior within the network, further enhancing gel structure and protein solubility, which are essential for desirable textural attributes (Ran et al., 2022b; Zhu et al., 2024). Among the plant-based formulations, PBS1 exhibited significantly lower pH value compared to the other treatments, which may be attributed to the higher content of acidic amino acids in SPI. However, the high pH may have implications for consumer safety and product preservation, as excessive alkalinity can affect microbial stability and shelf-life. Furthermore, it may influence sensory acceptability, potentially imparting a bitter taste. Therefore, formulation adjustments and careful monitoring of pH are essential to balance functional benefits with consumer safety and acceptability.

### 3.6 Scanning electron microscopy (SEM)

**Fig 2:**
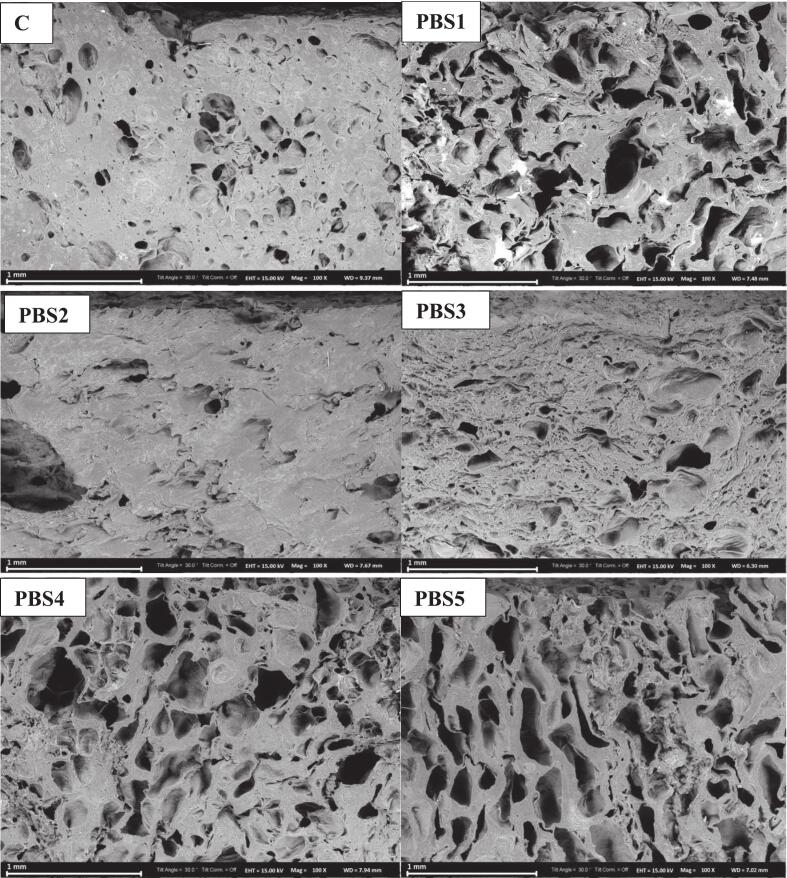

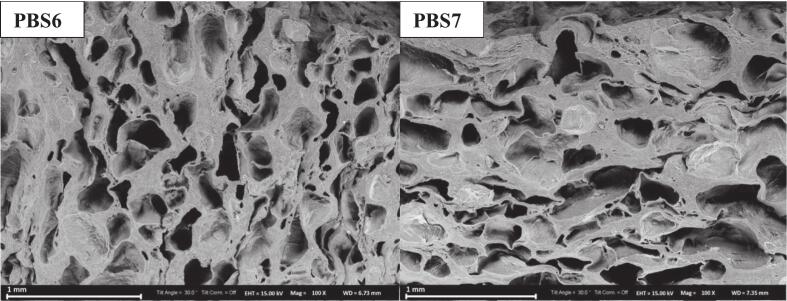
Cross-sectional SEM micrograph of surimi (control) and plant-based surimi. PBS1: 9% SPI; PBS2: 9% PPI; PBS3: 4.5% SPI + 4.5% PPI; PBS4: 9% MBPI; PBS5: 4.5% SPI + 4.5% MBPI; PBS6: 4.5% PPI + 4.5% MBPI; PBS7: 3% SPI + 3% PPI + 3% MBPI.

The microstructure of surimi plays a crucial role in determining its gelation properties, textural strength, and overall structural integrity. Microstructural analysis revealed that all samples developed a network structure, enabling the gels to display elastic properties. Upon heating, protein chains unfolded and subsequently aggregated, forming a three-dimensional gel network. The control sample exhibited small, uniformly distributed pores within its gel matrix (Fig. 2), resulting in a compact and well-organized structure. These findings are consistent with the observations reported by Ran et al. (2022b) for surimi fish balls.

For the PBS samples, the network structures differed depending on the type of protein used and their blending ratios. Except for PBS2 and PBS3, all other formulations exhibited large, irregular pores, resulting in a looser and softer gel structure. PBS2 exhibited the closest resemblance to the control, with a compact and continuous network with fewer large pores. This suggests that pea protein effectively formed strong gel networks. This observation aligns with Zhang et al., (2022), who found that pea protein forms phase-separated fibrous networks that improve gel strength and elasticity, making it a suitable alternative for seafood analogs.

The structural properties of PBS1 (soy protein-based surimi) revealed a moderately compact network, but with visible cracks and discontinuities, indicating incomplete protein gelation and lower cohesion compared to pea protein-based formulations. This correlates with previous studies on soy protein gelation, which highlight that soy proteins form moderately strong networks, but their ability to generate a homogenous structure is lower compared to fish-based myofibrillar proteins (Zhou et al., 2017). However, when soy protein was blended with pea protein in PBS3, the microstructure was significantly improved, with reduced porosity and a more cohesive network, confirming the benefits of blending different protein sources to improve gel integrity.

The PBS7 formulation, which incorporated all three protein sources (soy, pea, and mung bean), displayed an intermediate structure, with moderate porosity and partial aggregation. While the network appeared more compact compared to PBS6 and PBS4, it still lacked the level of cohesion observed in PBS2 or the control. The findings suggest that while blending different plant proteins can enhance gelation properties, optimizing protein ratios and modifying processing conditions is necessary to achieve superior structural integrity (Plattner et al., 2024).

### 3.7 Texture profile analysis

Texture is a fundamental characteristic in surimi-based products, influencing consumer acceptability and processing properties. Protein plays a crucial role in contributing to the hardness, chewiness, and overall mouthfeel of surimi products. As shown in Table 2, PBS2 exhibited the highest values for hardness, resilience, gumminess, and chewiness (P < 0.05), followed by the control and PBS1, while PBS4 and PBS6 showed the lowest values. These differences can be explained mechanistically by the intrinsic properties of the protein sources. Soy protein has higher cysteine content and unfolds readily during thermal treatment, promoting disulfide bond formation and creating a dense, cohesive gel network. Pea protein forms moderate gels through hydrophobic interactions and hydrogen bonding, while mung bean protein has lower sulfhydryl content, limiting disulfide cross-linking and network formation, resulting in weaker gels. In contrast, blending proteins at different ratios can reduce hardness. Guidi, Formica, and Denkel (2022) investigated the gel properties of hemp, pea, and lentil proteins both individually and in binary mixtures. They found that the gel strength of hemp protein decreased significantly when 25% of a second protein was introduced. This reduction was attributed to a dilution effect and the lack of ability to form a unified gel network. However, at higher blending ratios (50% and 75% of lentil and pea proteins, respectively), some level of protein–protein interaction seemed to occur. While the gel strength continued to decline with increasing hemp protein content, the pattern did not suggest a simple dilution effect, indicating that subfractions of proteins from different sources may interact to form a combined gel network. The effectiveness of such a network appears to be highly dependent on the mixing ratios. Similarly, in our study, protein blends in a 50:50 ratio exhibited lower hardness, while more balanced combinations improved gel strength. Additionally, the inclusion of oil can alter the mechanical properties of the protein matrix, affecting the product’s toughness and structural integrity. The type of oil used and its interaction with the protein source are critical considerations. In our previous study, incorporating an oleogel made from β-sitosterol and fish oil into fish balls significantly increased hardness. This enhancement was attributed to the formation of a three-dimensional gel-like network structure. β-sitosterol, when dispersed in edible oils, forms self-assembled fibrillar networks that entangle and trap oil droplets, resulting in a structured gel. This network reinforces the core structure and contributes to improved mechanical strength (Islam et al., 2024). Additionally, the lower hardness in control could be due to the absence of KGM. In a study conducted by Odelli et al. (2024), the effect of KGM addition on pea protein hydrogel was investigated. Texture analysis confirmed that KGM significantly influenced the texture of the hydrogels, with the highest KGM concentration (1.5%) resulting in the greatest hardness value of 1.34 N. This may be attributed to the removal of acetyl groups through deacetylation, which transforms KGM from a semi-crimped to a self-crimped state. This structural change promotes self-aggregation and the formation of three-dimensional networks capable of trapping water, thereby creating a more rigid and compact gel structure (Wang et al., 2017). The increased chewiness also could be due to KGM long-chain polysaccharide structure, which can interconnect the protein network and enhance the strength of the composite gel.

**Table 2:** Texture Profile Analysis of surimi (control) and plant-based surimi. Data are given as mean values ± standard deviation (n=3). Different letters within the same column indicate significant differences (Tukey’s Test, α = 0.05) between mean values.

Samples	Hardness	Resilience	Springiness	Gumminess	Chewiness
**PBS1**	3193.62±24.45^B^	32.03±0.93^B^	74.88±0.25^C^	1986.39±68.43^B^	1487.59±56.30^B^
**PBS2**	3781.11±144.26^A^	37.19±1.63^A^	85.06±1.18^B^	2477.52±126.90^A^	2106.37±78.31^A^
**PBS3**	1627.70±20.30^DE^	31.94±0.66^B^	75.29±0.09^C^	1100.07±5.98^D^	828.21±5.51^D^
**PBS4**	1454.40±30.96^EF^	27.68±0.60^C^	69.90±0.79^E^	903.92±30.77^E^	631.97±26.56^E^
**PBS5**	1717.94±70.53^D^	28.47±0.65^C^	70.79±1.74^DE^	1080.80±47.81^D^	765.16±42.05^D^
**PBS6**	1319.60±8.87^F^	27.42±0.26^C^	68.73±0.57^E^	821.86±3.56^E^	564.82±2.27^E^
**PBS7**	2733.61±33.61^C^	29.12±0.94^BC^	72.38±0.13^D^	1679.36±18.46^C^	1215.43±12.09^C^
**C**	1617.87±34.64^DE^	31.68±1.70^B^	90.84±0.16^A^	2328.21±35.10^A^	2147.66±43.03^A^

PBS1: 9% SPI; PBS2: 9% PPI; PBS3: 4.5% SPI + 4.5% PPI; PBS4: 9% MBPI; PBS5: 4.5% SPI + 4.5% MBPI; PBS6: 4.5% PPI + 4.5% MBPI; PBS7: 3% SPI + 3% PPI + 3% MBPI.

Scanning electron microscopy (SEM) images further revealed that PBS2 had a smooth, dense microstructure, whereas PBS1 showed a more porous network reflects a loose, more elastic network, leading to reduced hardness. Additionally, formulations incorporating mung bean protein, particularly PBS4 and PBS6, exhibited the lowest gel strength despite comparable moisture contents. This may be attributed to the inherently weaker gelation behavior of mung bean protein, which is characterized by a lower sulfhydryl group content and reduced capacity for disulfide bond formation.

Overall, the texture of plant-based surimi is governed by a complex interplay of protein chemistry, polysaccharide interactions, oil incorporation, water binding, and processing conditions. Understanding these mechanisms provides insight into how protein selection, blending ratios, and formulation strategies can be optimized to achieve desirable mechanical and sensory properties in plant-based surimi.

### 3.8 Viscoelastic properties

**Fig 3:**
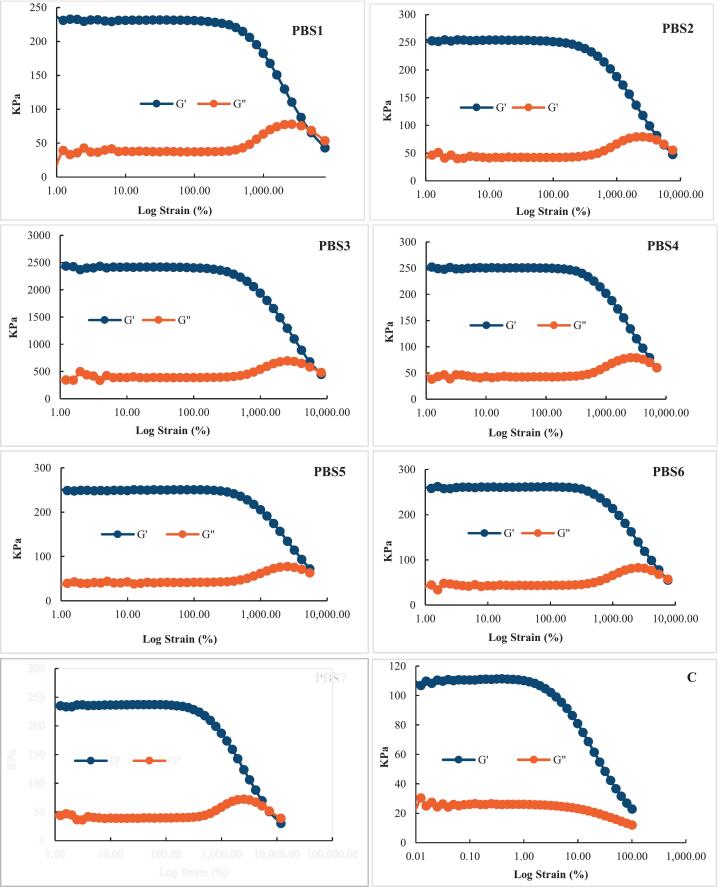
Oscillation strain of surimi (control) and plant-based surimi. PBS1: 9% SPI; PBS2: 9% PPI; PBS3: 4.5% SPI + 4.5% PPI; PBS4: 9% MBPI; PBS5: 4.5% SPI + 4.5% MBPI; PBS6: 4.5% PPI + 4.5% MBPI; PBS7: 3% SPI + 3% PPI + 3% MBPI.

The viscoelastic properties of PBS formulations and the control were evaluated using a strain sweep test to gain further insights into their structural behavior under deformation (Figure 3). The loss modulus (G") represents the viscous behavior of the network, while the storage modulus (G') reflects the elastic, solid-like characteristics (Yuliarti, Kovis, & Yi, 2021). As shown in Figure 3, at relatively small strains, G′ consistently exceeded G′′ across all samples, indicating dominant elastic behavior. Both modulus values remained nearly constant within this range, further supporting the presence of the LVR. At higher strain levels, G' and G" intersected, indicating structural breakdown and a shift toward viscous behavior due to the dominance of G". Notably, the intersection point varied among PBS formulations, likely due to differences in the behavior of SPI, PPI, and MBPI. Similar trends were reported by Ran et al. (2022a), suggesting enhanced network coupling and improved gel quality in PBS formulations.

Similarly, the G' of the control surimi decreased with increasing strain. However, unlike the PBS formulations, G' and G” did not intersect; G' remained dominant, indicating the elastic nature of the control. This behavior may be attributed to the absence of KGM in control formulation. In a previous study by Iglesias-Otero, Borderías, and Tovar (2010), the effects of KGM dispersion in water (10 wt.%) at various concentrations (0%, 1%, 5%, and 10%) under alkaline pH conditions were examined on the viscoelastic properties of giant squid surimi and the gels formed under different thermal treatments. The study found that samples without KGM (0%) had significantly lower viscoelastic properties compared to those containing KGM. However, no significant differences were observed among the 1%, 5%, and 10% KGM groups, indicating that 1% was the minimal effective concentration. These results are in support of SEM and textural results.

### 3.9 Thermal properties

**Fig 4:**
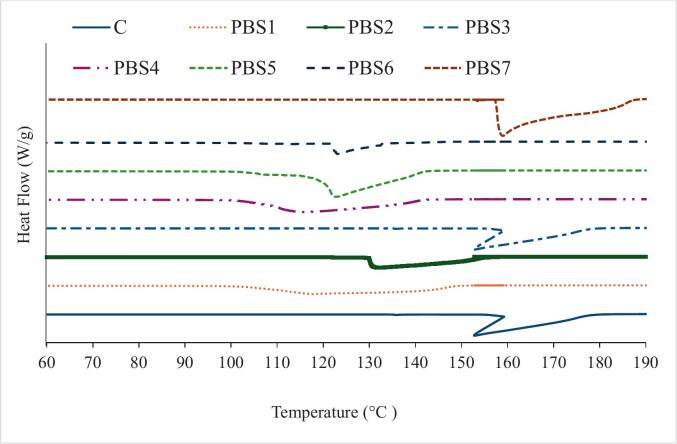
Differential scanning calorimetry (DSC) of surimi (control) and plant-based surimi PBS1: 9% SPI; PBS2: 9% PPI; PBS3: 4.5% SPI + 4.5% PPI; PBS4: 9% MBPI; PBS5: 4.5% SPI + 4.5% MBPI; PBS6: 4.5% PPI + 4.5% MBPI; PBS7: 3% SPI + 3% PPI + 3% MBPI.

Differential scanning calorimetry (DSC) is commonly used to investigate the thermal behavior of seafood proteins and other protein sources following various treatments. The process of thermal unfolding—an initial stage of protein denaturation—requires heat input to disrupt the hydrogen bonds that maintain the protein’s native three-dimensional structure, resulting in an endothermic reaction. All PBS formulations in this study exhibited a single peak at 114 °C, 128°C, 152°C, 110°C, 111°C, 118°C, and 150°C for PBS1 -7, respectively (Fig. 4 and Table 3). The presence of only one peak may suggest that the commercial protein isolates were already partially or fully denatured. Shand, Ya, Pietrasik, and Wanasundara (2007) also investigated the thermal behavior of commercial PPI and reported the absence of additional endothermic peaks, which they attributed to the potential denaturation of major proteins in the product. Likewise, Arntfield and Murray (1981) noted that the lack of a thermal endotherm is often indicative of denatured proteins, especially when the native form typically shows a distinct peak under standard analytical conditions. They also found no distinguishable thermal transition peaks in the thermogram of commercial SPI, further indicating protein denaturation in the product. Additionally, the high peak temperatures observed in our study may be attributed to the incorporation of KGM. Zhang et al. (2024) investigated the gel properties of hydrogels formed through interactions between SPI and KGM, reporting an increase in the Tmax of the composite gel from 140.06 to 150.02 °C as KGM concentration increased. This suggests that KGM interacts with SPI, enhancing hydrogen bonding within the gel’s crosslinked network. As a result, the thermal stability of the gel improved, requiring more energy to disrupt its molecular structure. Similarly, Park, Kim, and Shin (2018) found that the incorporation of polysaccharides enhanced the thermal stability of fucoidan-β-lactoglobulin gels by contributing to the formation of a more stable spatial structure.

**Table 3:** Thermal characteristics of surimi (control) and plant-based surimi. Data are presented as mean ± standard deviation (n=3). Different letters within the same column indicate significant differences between mean values (Tukey’s test, α = 0.05).

Treatment	Tonset (^◦^C)	Tpeak (^◦^C)	Tendset (^◦^C)	Enthalpy ΔHm (J/g)
**PBS1**	103.74±1.11^E^	114.44±1.09^C^	102.31±0.95^D^	2227.1±0.84^C^
**PBS2**	128.81±1.34^C^	128.89±1.01^B^	128.92±1.82^B^	1501.4±1.98^F^
**PBS3**	158.31±1.12^A^	152.85±1.54^A^	157.60±1.44^A^	1893.5±1.19^E^
**PBS4**	105.02±0.99^E^	110.75±1.13^C^	104.61±1.32^D^	2514.3±1.87^B^
**PBS5**	115.40±0.97^D^	111.97±1.03^C^	115.51±1.66^C^	3265.2±1.79^A^
**PBS6**	120.26±1.46^CD^	118.30±1.39^C^	120.94±1.83^C^	801.41±1.14^G^
**PBS7**	162.65±1.29^A^	150.56±1.99^A^	155.43±1.23^A^	3171.0±2.01^A^
**C**	137.18±0.93^B^	150.04±1.36^A^	137.49±1.00^B^	2065.5±1.67^D^

PBS1: 9% SPI; PBS2: 9% PPI; PBS3: 4.5% SPI + 4.5% PPI; PBS4: 9% MBPI; PBS5: 4.5% SPI + 4.5% MBPI; PBS6: 4.5% PPI + 4.5% MBPI; PBS7: 3% SPI + 3% PPI + 3% MBPI.

When two different polymers are combined, their interactions can lead to either associative (synergistic) or segregative (antagonistic) effects. A synergistic effect typically arises when the components are structurally compatible or when one acts as an active filler within the matrix. Conversely, structural incompatibility may result in segregative behavior. For instance, in whey protein isolate/corn starch gels, disrupted starch granules can act as inactive fillers, weakening the structure or causing phase separation (Li, Wei, Fang, Zhang, & Zhang, 2014). In this study, mixing SPI and PPI resulted in a thermal transition peak at a higher temperature (PBS3), suggesting potential synergistic interactions. The higher transition peaks observed in the pea–soy (PPI–SPI) blends may be attributed to the complementary protein structures and functional properties of these isolates, which favor synergistic interactions. Soy protein isolate (SPI) is rich in glycinin and β-conglycinin, which form compact, globular structures stabilized by hydrogen bonds and disulfide linkages. Pea protein isolate (PPI), on the other hand, contains legumin and vicilin, which can unfold more readily during heating and interact with SPI molecules through hydrogen bonding and hydrophobic interactions. When blended, these proteins are likely forming an integrated network with enhanced intermolecular interactions, resulting in increased structural order and, consequently, higher thermal transition temperatures. This synergistic behavior has been reported in mixed protein systems where complementary amino acid compositions promote stronger gel matrices and improved thermostability (Li et al., 2014; Zhang et al., 2024).

However, when SPI was blended with MBPI or PPI with MBPI, the transition peaks occurred at lower temperatures. MBPI consists mainly of 8S globulin, which exhibits weaker hydrophobic interactions and lower sulfhydryl content compared to SPI and PPI. These structural characteristics reduce its ability to form strong intermolecular crosslinks during heating, leading to weaker gel networks and lower transition peaks. Furthermore, the relatively lower denaturation temperature of MBPI indicates reduced structural compactness and fewer stabilizing intramolecular interactions. When blended with either SPI or PPI, MBPI may act as a diluting or segregative component, disrupting the cohesive protein network and decreasing the energy required for denaturation.

Overall, the higher transition peaks in the pea–soy blends reflect synergistic protein–protein interactions and enhanced thermal stability, while the lower peaks in mung bean–based blends result from limited compatibility and weaker molecular interactions within the gel matrix. Notably, when a balanced ratio of SPI, PPI, and MBPI was used in PBS7, a significantly higher transition peak was observed, further supporting the presence of a synergistic effect. The observed thermal transitions and rheological properties of the plant-based surimi formulations can be attributed to protein denaturation, the availability of free sulfhydryl groups, and protein–polysaccharide interactions. Increased denaturation temperatures and storage modulus (G’) reflect stronger protein unfolding and reorganization, which facilitates the formation of a stable gel network. Free sulfhydryl groups promote disulfide bond formation, further reinforcing the gel structure, while interactions between proteins and KGM enhance network cohesion, water-holding capacity, and viscoelastic properties, contributing to improved texture and thermal stability.

The total heat energy, or enthalpy (ΔH), required for an endothermic reaction is represented by the area under the DSC peak. Enthalpy (ΔH) serves as an indicator of the ordered conformation of proteins, with the “net” ΔH reflecting the combined effects of endothermic events—such as the disruption of hydrogen bonds—and exothermic processes, such as protein aggregation via hydrophobic interactions. Odelli et al. (2024) reported lower ΔH values for PPI samples compared to those observed in our study. This discrepancy could be attributed to the lower concentrations of KGM used in their formulations. They proposed that KGM molecules may interact with proteins in a way that affects the structural integrity, packing density, and thermal stability of the protein matrix. At higher KGM concentrations, steric repulsion between polymers may become more pronounced, potentially accelerating protein thermal transitions by acting in a plasticizing manner (Tahergorabi, Sivanandan and Jaczynski, 2012).

Surimi is primarily composed of myofibrillar proteins, as it is produced through repeated washing of mechanically deboned and minced fish muscle to remove impurities such as sarcoplasmic proteins. DSC reveals endothermic peaks that correspond to the heat absorbed during the unfolding of protein structures as they transition to a denatured state. Typically, two distinct peaks are observed: the first is attributed to myosin denaturation, and the second to actin, as reported in our previous study (Tahergorabi & Jaczynski, 2012). This dual-peak pattern is expected in samples with similar protein composition and gelation properties. However, in our control sample—as with PBS formulations particularly PBS7 and PBS3—only a single peak was detected at approximately 150 °C, with no significant differences among them. The elevated endothermic transition temperatures observed in the control samples may be attributed to the presence of tapioca starch. A similar trend was reported by Kong et al. (2016) in Alaska pollock surimi supplemented with starch. This increase is likely associated with starch gelatinization. It is suggested that the thermal transition peak of starch overlaps with that of surimi proteins, resulting in a combined peak. Consequently, the total area under this peak reflects the cumulative enthalpy changes from both protein denaturation and starch gelatinization, potentially leading to higher endothermic transition temperatures. These findings are consistent with the results observed in the TPA and rheology analyses.

### 3.10 Amino acids profile

The evaluation of the amino acid profiles presented in Table 4 highlights the nutritional differences between fish-based surimi and various formulations of plant-based surimi. Increasing protein levels can improve the nutritional profile of meat alternatives, making them more comparable to animal-based meats in terms of protein content. However, the focus should not be solely on protein quantity; protein quality, digestibility, and amino acid composition are equally important factors in assessing the overall nutritional value of meat alternatives (Singh & Sit, 2022). All treatments contained EAAs; however, the control surimi demonstrated significantly higher levels of EAA overall followed by PBS1 and PBS2. Soy protein is a well-established source of complete protein, providing all nine EAA required by the human body. Pea protein also has a well-balanced amino acid profile, notably rich in lysine, arginine, and branched-chain amino acids (BCAAs) such as leucine, isoleucine, and valine (Lu, He, Zhang, & Bing, 2020).  The analysis indicated that the threonine content in the control was 6.83 ± 0.1, significantly higher than the PBS treatments. PBS1 was the closest to control with a threonine level of 3.4 ± 0.1, although still significantly lower. Threonine is crucial for protein synthesis, immune function, and maintaining a healthy gut, this amino acid is notably limited in many plant protein sources, which often require careful combinations to meet dietary needs effectively (Pinckaers, Trommelen, Snijders, & van Loon, 2021; Tang, Tan, Ma, & Ma, 2021). Significant differences were also observed in valine content, with control yielding 7.25 ± 0.1, while values for the PBS options varied. Specifically, PBS4 was significantly lower at 3.85 ± 0.1. However, mung bean protein’s digestible indispensable amino acid score (DIAAS) is 86, which is higher than that of pea protein (70) but slightly lower than soybean protein (91) (McClements & Grossmann, 2021). Valine is essential for muscle metabolism and tissue repair, and its deficiency can adversely affect performance and recovery, especially for active individuals (Sharma et al., 2023). The evaluation of isoleucine and leucine illustrates a further disparity, with control exhibiting significantly higher amounts (7.19 ± 0.1 and 12.5 ± 0.1, respectively). These BCAAs are vital for muscle recovery and energy production, emphasizing the role of fish-derived proteins in athletic and health-conscious diets (Wolfe, 2017). The superiority of control continued with levels of methionine and tryptophan, recorded at 5.02 ± 0.1 and 1.53 ± 0.1, which were significantly higher than those observed in the plant-based treatments. Methionine plays a critical role in various metabolic processes, including the synthesis of other amino acids and the production of important molecules like S-adenosylmethionine, which is involved in numerous biological functions (Abotsi, Panagodage, & English, 2024). Moreover, tryptophan serves as a precursor for serotonin and melatonin, which are important for mood regulation and sleep. While fish-based surimi provides a robust source of EAAs necessary for various physiological functions, PBS formulations exhibit variability in their amino acid profiles. The EAAs in the current study were higher than those reported by Zhao et al. (2023), but lower than the findings of Cañada Millán, Hurtado Sarabia, Ramos Carrera, and Quevedo Santos (2021) The amino acid profile of surimi can vary significantly due to differences in processing methods, fish species, and the geographic location where the fish were harvested (Yin & Park, 2023).

**Table 4:** Amino acid profile of surimi (control) and plant-based surimi. Data are presented as mean ± standard deviation (n=3). Different letters within the same row indicate significant differences between mean values (Tukey’s test, α = 0.05).

Amino acids (g/100g)	Treatments							
	PBS1	PBS2	PBS3	PBS4	PBS5	PBS6	PBS7	C
Aspartic Acid	10.4±0.1^B^	9.79±0.1^C^	9.05±0.1^D^	8.46±0.1^E^	9.88±0.1^C^	9.63±0.1^C^	9.23±0.1^D^	15.5±0.1^A^
Threonine	3.4±0.1^B^	3.11±0.1^C^	2.88±0.1^CD^	2.23±0.1^E^	2.92±0.1^CD^	2.83±0.1^CD^	2.71±0.1^D^	6.83±0.1^A^
Serine	4.7±0.1^B^	4.31±0.1^CD^	4.04±0.1^DE^	3.92±0.1^E^	4.49±0.1^BC^	4.32±0.1^CD^	4.1±0.1^DE^	6.45±0.1^A^
Glutamic Acid	17.1±0.1^B^	14.2±0.1^D^	14.3±0.1^D^	12.5±0.1^F^	15.6±0.1^C^	14.4±0.1^D^	13.9±0.1^E^	25±0.1^A^
Proline	4.66±0.1^A^	3.66±0.1^C^	3.77±0.1^C^	3.2±0.1^D^	4.12±0.1^B^	3.68±0.1^C^	1.71±0.1^E^	4.67±0.1^A^
Glycine	3.7±0.1^B^	3.41±0.1^C^	3.2±0.1^CD^	2.46±0.1^E^	3.24±0.1^CD^	3.08±0.1^D^	2.97±0.1^D^	5.08±0.1^A^
Alanine	3.84±0.1^B^	3.66±0.1^BC^	3.49±0.1^CD^	3.03±0.1^E^	3.49±0.1^CD^	3.56±0.1^BCD^	3.36±0.1^D^	8.53±0.1^A^
Valine	4.22±0.1^B^	4.2±0.1^B^	3.77±0.1^C^	3.85±0.1^C^	4.25±0.1^B^	4.2±0.1^B^	3.98±0.1^BC^	7.25±0.1^A^
Isoleucine	4.25±0.1^B^	3.98±0.1^BC^	3.68±0.1^D^	3.32±0.1^E^	3.99±0.1^BC^	3.85±0.1^CD^	3.71±0.1^CD^	7.19±0.1^A^
Leucine	7.15±0.1^B^	7.03±0.1^B^	6.32±0.1^CD^	6.11±0.1^D^	7.04±0.1^B^	6.88±0.1^B^	6.55±0.1^C^	12.5±0.1^A^
Tyrosine	3.53±0.1^B^	3.3±0.1^BC^	3.06±0.1^CDE^	2.41±0.1^F^	3.12±0.1^CD^	2.93±0.1^DE^	2.82±0.1^E^	5.89±0.1^A^
Phenylalanine	4.85±0.1^BC^	4.61±0.1^C^	4.19±0.1^D^	4.71±0.1^C^	5.1±0.1^B^	4.89±0.1^BC^	4.61±0.1^C^	5.44±0.1^A^
Lysine	5.34±0.1^C^	5.78±0.1^B^	5.03±0.1^D^	4.77±0.1^DE^	5.53±0.1^BC^	5.51±0.1^BC^	4.62±0.1^E^	14±0.1^A^
Histidine	2.24±0.1^B^	2.02±0.1^BC^	1.93±0.1^C^	2.04±0.1^BC^	2.27±0.1^B^	2.15±0.1^BC^	2.02±0.1^BC^	3±0.1^A^
Arginine	7.08±0.1^C^	7.43±0.1^B^	6.51±0.1^D^	5.33±0.1^F^	6.48±0.1^DE^	6.54±0.1^D^	6.2±0.1^E^	10.1±0.1^A^
Cystine	0.91±0.1^B^	0.62±0.1^CD^	0.79±0.1^BC^	0.1±0.1^E^	0.45±0.1^D^	0.39±0.1^D^	0.48±0.1^D^	1.54±0.1^A^
Methionine	1.18±0.1^B^	0.95±0.1^B^	0.99±0.1^B^	1.02±0.1^B^	1.17±0.1^B^	1.06±0.1^B^	1±0.1^B^	5.02±0.1^A^
Tryptophan	1.09±0.1^B^	0.77±0.1^CD^	0.90±0.1^BCD^	0.66±0.1^D^	0.95±0.1^BC^	0.74±0.1^CD^	0.86±0.1^BCD^	1.53±0.1^A^

PBS1: 9% SPI; PBS2: 9% PPI; PBS3: 4.5% SPI + 4.5% PPI; PBS4: 9% MBPI; PBS5: 4.5% SPI + 4.5% MBPI; PBS6: 4.5% PPI + 4.5% MBPI; PBS7: 3% SPI + 3% PPI + 3% MBPI.

When compared to adult RDA values for essential amino acids (FAO/WHO 2007), fish-based surimi (control) meets or exceeds the daily requirements for all EAAs, including lysine (14 g/100 g protein vs. RDA 45 mg/g protein), threonine (6.83 g/100 g vs. 23 mg/g), and methionine + cystine (6.56 g/100 g vs. 22 mg/g). In contrast, plant-based surimi formulations contain lower levels of some limiting amino acids; for example, lysine in PBS1–PBS7 ranges from 4.62–5.78 g/100 g protein, which is below the RDA, and methionine + cystine ranges from 0.49–1.18 g/100 g protein, also below the recommended level. Other EAAs in the PBS formulations are closer to or meet RDA values. This indicates that while plant-based surimi contributes important protein, complementary protein sources or fortification may be needed to fully satisfy dietary requirements.

### 3.11 Color

**Fig. 5:**
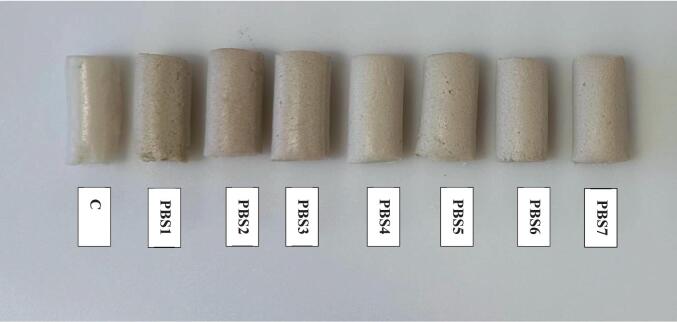
Visual appearance of surimi (control) and plant-based surimi. PBS1: 9% SPI; PBS2: 9% PPI; PBS3: 4.5% SPI + 4.5% PPI; PBS4: 9% MBPI; PBS5: 4.5% SPI + 4.5% MBPI; PBS6: 4.5% PPI + 4.5% MBPI; PBS7: 3% SPI + 3% PPI + 3% MBPI.

Visually, the control surimi made from Alaska pollock were glossier, firmer, and more translucent compared to the plant-based samples (Fig. 5). Among the formulations, the PBS2 and PBS4 most closely resemble the control in terms of smoothness, structural integrity, and uniform appearance. This suggests that PPI and MBPI may replicate the texture and appearance of traditional surimi. The color of food products, especially surimi, is a major factor in shaping consumer perceptions of quality, taste, and freshness (Chehade, Angelino, Del Bo, Maggioni, Pellegrini, Riso, & Martini, 2025). As shown in Table 5, The fish-based surimi (control), had the highest whiteness index (79.98 ± 1.12). This was accompanied by a very low redness value (-2.70 ± 0.04), pointing to the absence of red tones and a more neutral white appearance. This outcome is expected, since traditional Alaska pollock surimi is known for its bright white color, achieved through the removal of natural pigments during processing. Chaijan, Panpipat, and Benjakul (2010) investigated color values of surimi prepared from three varieties of mackerel captured in Southern Thailand. The whiteness index values from their studies ranged between 50.56 and 59.14 were lower when compared with the level of whiteness index of fish-based surimi in our study. This is most likely due to the difference between species, as surimi made from different types of fish has different colors.

**Table 5:** Color changes of surimi (Control) and plant-based surimi. Data are given as mean values ± standard deviation (n=3). Different letters within the same column indicate significant differences (Tukey’s Test, α = 0.05) between mean values.

Samples	L*	a*	b*	WI
**PBS1**	73.75±1.75^B^	-0.45±0.04^D^	9.45±0.35^AB^	72.08±1.53^C^
**PBS2**	80.10±1.21^A^	0.21±0.04^A^	9.52±0.07^AB^	77.93±1.06^AB^
**PBS3**	73.54±2.64^B^	0.02±0.04^B^	9.68±0.10^A^	71.81±1.46^C^
**PBS4**	80.08±2.54^A^	-0.66±0.01^E^	9.01±0.12^ABC^	78.11±1.31^AB^
**PBS5**	77.20±0.66^AB^	-0.95±0.07^F^	9.41±0.30^AB^	75.31±0.56^ABC^
**PBS6**	74.07±0.92^B^	-0.19±0.03^C^	8.39±0.16^C^	72.74±0.87^C^
**PBS7**	76.55±3.21^AB^	-0.53±0.04^DE^	8.91±0.44^BC^	74.88±1.14^BC^
**C**	80.27±1.17^A^	-2.70±0.04^G^	2.05±0.18^D^	79.98±0.1.12^A^

PBS1: 9% SPI; PBS2: 9% PPI; PBS3: 4.5% SPI + 4.5% PPI; PBS4: 9% MBPI; PBS5: 4.5% SPI + 4.5% MBPI; PBS6: 4.5% PPI + 4.5% MBPI; PBS7: 3% SPI + 3% PPI + 3% MBPI.

Lightness is considered a key quality marker in surimi and seafood alternatives, as consumers tend to associate whiter products with better quality and freshness (Choi et al., 2023). Among the plant-based samples, PBS2 and PBS4 exhibited the highest lightness values (80.10 ± 1.21 and 80.08 ± 2.54, respectively), which were not significantly different from the control. Several mechanistic factors likely explain the observed color, gloss, and translucency differences such as intrinsic pigments and residual chromophores. Plant protein isolates often retain trace pigments (carotenoids, flavonoids, chlorophyll degradation products) or bound phenolic compounds that impart a yellowish or off-white hue; the degree of pigment removal during protein isolation varies by source. For instance, **s**oy protein often has slightly yellowish hue due to its natural composition, which can impact on the overall color of products made from it. In contrast, pea and mung bean proteins typically have a more neutral or lighter color, contributing to the observed similarity in lightness (Sha & Xiong, 2020). Moreover, particle size, porosity, and network density control how light is scattered or transmitted: denser, smoother gels (finer protein network, low porosity) scatter less light and appear glossier and more translucent (as seen for PBS2), whereas porous, heterogeneous networks scatter lighter and appear matte or more opaque. Additionally, in this study, TiO_2_ was added to the PBS samples to increase brightness and whiteness of plant-based surimi. TiO_2_ is a whitening agent that brightens and enhances the white color of foods by effectively scattering light waves, thereby improving consumer acceptance of meat analogs. Its effectiveness in enhancing the visual appearance of plant-based products was previously demonstrated by Liao, Li, and Tjong (2020) in formulations of plant-based chicken. However, their redness values (ranging from 0.21 ± 0.04 to -0.66 ± 0.01) were notably higher than the control’s, showing a slight shift away from pure white. Even so, the whiteness index scores of PBS2 (77.93 ± 1.06) and PBS4 (78.11 ± 1.31) were close to the control, suggesting that these two samples visually resemble traditional fish surimi more than the other plant-based versions.

The yellowness values across the PBS samples were relatively consistent, ranging from 8.39 ± 0.16 to 9.68 ± 0.10. In contrast, the fish-based control had a much lower yellowness value (2.05 ± 0.18), making it significantly less yellow than any of the PBS options. This stronger yellow hue in the PBS samples may be due to the use of oleogel and the presence of pigments in certain plant proteins, such as soy. Moreover, heating and interactions between reducing sugars and amino groups can produce brown/yellow chromophores; formulation sugar content, thermal profile, and protein composition (lysine content) influence the extent of non-enzymatic browning. These mechanisms are consistent with our observations: PBS2 and PBS4’s higher lightness and whiteness correlate with smoother, denser microstructure (SEM) and lower residual pigment load, while higher yellowness across PBS samples aligns with the presence of plant pigments, oleogel composition, or minor Maillard reaction products. These findings underline how critical it is to choose the right protein sources and blends when developing plant-based surimi, as visual appeal plays a major role in consumer acceptance.

### 3.12 Sensory analysis

**Fig 6:**
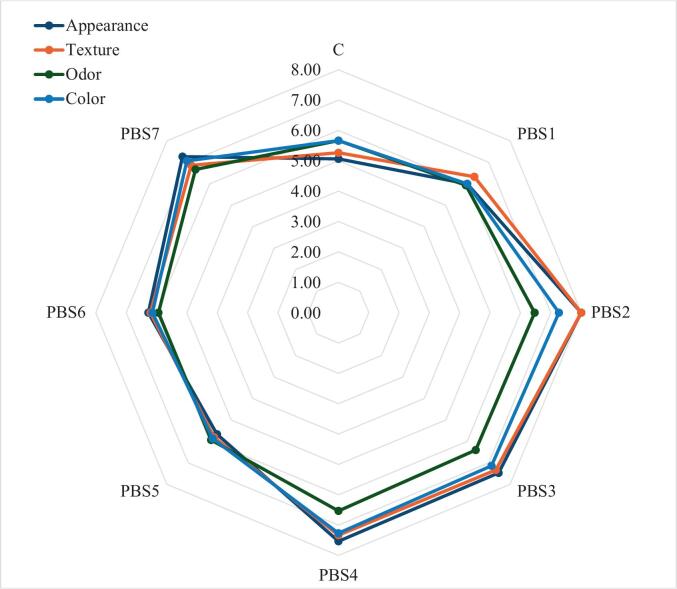
Sensory Evaluation of plant-based surimi and surimi (control). PBS1: 9% SPI; PBS2: 9% PPI; PBS3: 4.5% SPI + 4.5% PPI; PBS4: 9% MBPI; PBS5: 4.5% SPI + 4.5% MBPI; PBS6: 4.5% PPI + 4.5% MBPI; PBS7: 3% SPI + 3% PPI + 3% MBPI.

Sensory evaluation is essential for objectively measuring sensory attributes, including appearance, texture, odor, and color, to determine consumer acceptance and preference. In this study, scores equal to or higher than 7 were considered acceptable. As illustrated in Fig. 6, PBS2 received the highest appearance score of 8 ± 1.46, whereas the control recorded the lowest score of 5.07 ± 2.01, deemed unacceptable. The remaining PBS samples scored between 5.67 ± 2.02 and 7.53 ± 1.41, suggesting that panelists moderately favored the plant-based samples. Texture scores followed a similar pattern, with PBS2 again achieving the highest score of 8 ± 1.13, while the control had the lowest texture score of 5.27 ± 2.00. Color scores for PBS samples ranged from 6 ± 1.73 (PBS1) to 7.27 ± 1.16 (PBS4), with the control slightly exceeding a score of 5. In terms of odor, all samples scored comparably, ranging from 5.67 ± 1.95 (control) to 6.67 ± 1.4 (PBS7). Overall, PBS samples consistently received higher scores compared to the control. Notably, PBS2, PBS3, PBS4, and PBS7 achieved acceptable scores (above 7), indicating enhanced acceptability and preference among panelists. These findings confirm that consumers prefer plant-based surimi over the fish-based control, with PBS2 standing out particularly in terms of appearance and texture attributes. However, it should be noted that the sensory panel size was limited to 10 semi-trained individuals, which may restrict the representativeness and generalizability of the results. Therefore, while the findings provide useful preliminary insights, larger and more diverse panels will be needed in future studies to validate consumer acceptability and market potential of the plant-based surimi formulations.

## 4 Conclusions

This study provides detailed insights into the development and characterization of plant-based surimi (PBS) as a sustainable alternative to traditional fish-based surimi. The protein composition and blending strategies critically influenced the physicochemical, textural, rheological, nutritional, and structural properties of PBS formulations. Mechanistically, the superior performance of PBS2 (pea protein-based) can be attributed to its strong gelation ability, effective protein–protein interactions, and higher moisture content, resulting in a dense, cohesive gel network and amino acid profile comparable to conventional surimi. In contrast, mung bean protein-based formulations (PBS4 and PBS6) exhibited weaker gel networks and higher porosity due to lower sulfhydryl content and reduced capacity for disulfide bond formation, underscoring the need for formulation optimization. Blended formulations, particularly PBS7 (soy-pea-mung bean), demonstrated enhanced viscoelastic and thermal stability, evidenced by higher storage modulus and denaturation temperatures, reflecting synergistic protein–protein and protein–polysaccharide interactions. Collectively, these findings highlight the mechanistic roles of protein selection, strategic blending, and processing conditions in determining gel structure, thermal behavior, and sensory attributes. Future research should explore enzymatic crosslinking, incorporation of complementary hydrocolloids, and alternative structuring strategies to further enhance gel strength, nutritional quality, and consumer acceptance of plant-based surimi. These insights provide a scientific foundation for the rational design of high-quality plant-based seafood analogs with optimized texture, nutrition, and functional properties.

## CRediT authorship contribution statement

**Shahriyar Valizadeh:** Writing – original draft, Visualization, Formal analysis. **Armin Mirzapour-Kouhdasht:** Writing – review & editing. **Mahagani Lasciers:** Formal analysis. **Reza Tahergorabi:** Writing – review & editing, Supervision, Funding acquisition, Conceptualization.

## Funding

This work was supported by the USDA National Institute of Food and Agriculture, 1890 Capacity Building Grants Program, award # 2023-38821-39980.

## Declaration of competing interest

The authors declare that they have no known competing financial interests or personal relationships that could have appeared to influence the work reported in this paper.

## Data Availability

The data are available within the manuscript
